# On the Diagnosis of Dislocations and Fractures of the Upper Extremity of the Humerus

**Published:** 1829-01-01

**Authors:** 


### Diagnosis of Dislocations and Fractures of the Upper Extremity of the Humerus. By *M. Le Baron Dupuytren*.

[Reported by M. Marx.—Repertoire d'Anatomie.]

Fractures, of late years, have received much attention, but they never can be studied more than they deserve. Taking it all in all, the subject may be said to comprise within itself an epitome almost of the science of surgery; for the highest, the most curious, and most difficult questions, as well as the most homely and every-day matters of practice, fall within the limits of its wide domain. Far be it from us to enter on the discussion of so vast a topic, for even did we possess the ora centum—ferrea vox of the Mantuan Bard, we should shrink from the task in utter dismay. Our business at present is with only one division, and that a very small one, of the series of fractures and luxations to which the articular and osseous apparatus of the human frame is unfortunately liable. We are only in a corner, as it were, of the body, and yet in that corner, little as it is, how constantly ignorant men are blundering—how often do even the ablest go astray!

It is perfectly undeniable that fractures of the upper extremity of the humerus and dislocations of the head of that bone, especially dislocation into the axilla, are at times confounded with, and mistaken for, each other, even by those whose experience is great; we allude to our hospital surgeons. If mistakes then occur with those whose opportunities of practice are considerable, à fortiori, how much more frequent must they be amongst those who have few or no such opportunities, and in country practice in particular. The question indeed need not be begged, for the fact, as so put, is unhappily notorious. In some cases, they are not so many as we all might wish, a little diagnostic slip is not attended with very much practical error, or with any very mischievous effects. Here, however, we have no such loop-hole, nor dare we lay that anodyne unction to our souls. As M. Dupuytren justly observes, if luxation is taken for a fracture, reduction is seldom completely effected; and if fracture is taken for luxation, reduction is effected, it is true; but the parts are afterwards comparatively abandoned to themselves, the fracture is soon again displaced by the action of the muscles, and the patient is more or less crippled in the limb. So much for the mischiefs of erroneous diagnosis; let us now consider the point of diagnosis itself. The cases and remarks to follow must carry along with them a great, intrinsic, and practical importance, as coming avowedly from one, who stands at this moment, if not the highest, yet amongst the highest, and surpassed by none, of surgeons in the old world or the new. The name and authority of M. Dupuytren are passports to the attention of every practical man.

Every individual, says the Baron, affected with either dislocation or fracture of the upper extremity of the bone, has received a fall on that side of the body.* In the two cases, however, the position of the limb at the time of the accident differs, and furnishes the means of distinguishing, in general, the nature of the accident itself. If the arm was stretched out at the time, either forwards or backwards, in order to break the force of the fall, and displacement ensues, it depends on dislocation of the head of the humerus, without any fracture. On the contrary, if the arm was applied to the side or the chest at the instant of the accident, the hand being, for instance, in the breeches' pocket, the weight of the body tells on the projecting shoulder, and displacement is the consequence of fracture of the head or superior portion of the humerus. The patient cannot always describe the exact mode in which he met with the injury, and pain being referred both in fracture and luxation to the shoulder, he commonly, indeed almost always, believes that this was the part upon which he fell. The following circumstances will enable the surgeon to decide aright.

In dislocation, the fall has taken place on the palm of the hand, and this almost always tells its own tale, being soiled with dirt, or presenting ecchymoses and abrasions. In fracture, the fall having been upon the shoulder, the soft parts are contused, or the clothes in that situation shew traces of injury, whilst the palm of the hand is free from any marks. Again, in dislocation the pain, and ecchymosis, and engorgement, have a different seat from that which they occupy in fracture; being, in the former, on the inner or anterior part of the arm; in the latter, more immediately on the bruised part, the shoulder itself. Ecchymosis, too, is much more rare in dislocation than it is in fracture. There are other symptoms besides which assist diagnosis.

---

* This must be received with certain reservations. Thus a boy is holding a horse

Though, in both these cases, the deltoid is flattened and acromion prominent, with a hollow beneath it, and projection in the axilla, yet still, on a close examination, these symptoms, apparently the same, are found, in reality, to differ. In luxation, prominence of the acromion is greater, and the flattening of the deltoid more decided than in fracture; the muscle, in the latter case, appearing not so much flattened as shortened, and, as it were, swollen. In dislocation, the hollow beneath the acromion is deeper, and the projection in axillâ bolder, rounder, and more equal than in fracture; all these circumstances shewing that, in one case, the displacement is far more complete than in the other. Motion and crepitus are imperfect in luxation; both are obtained with facility in fracture. In dislocation, indeed, we try to move the limb in vain; for commonly the scapula moves with it, as much as if both were part and parcel of each other. In fracture, on the contrary, the motion, so far from being thus imperfect, is exaggerated at one particular point of the upper end of the bone, whilst crepitus is most distinctly felt, by seizing the elbow, and rotating the os humeri on its axis. Lastly, in dislocation it requires more force to replace the bone than in fracture, and less, when replaced, to retain it.

Fracture occasionally happens, without being attended by displacement, and is liable to be considered as merely a contusion. The crepitus and unnatural mobility on rotating the elbow, are the only means of detecting the accident; and here it must be borne in mind, that a violent contusion of the shoulder-joint will sometimes occasion a kind of crepitation or crackling, resulting from inflammation of the articulating surfaces, and deficient synovial secretion. Fifteen cases are appended, from the practice of the Hôtel Dieu, in illustration and confirmation of the foregoing remarks. They exemplify, in succession, fractures of the upper extremity and neck of the humerus, and dislocations of the head of the bone—fracture mistaken for luxation, and the latter mistaken for fracture—and, finally, fractures confounded with simple contusion. We do not mean to dwell with the same degree of minuteness upon all, though all possess many features of importance. The first we shall briefly notice, as presenting the clear and unadulterated symptoms of fracture of the upper extremity of the humerus, as well as the treatment employed for that accident at the Hôtel Dieu.

Marie-Susanne Fillet, ætatis 78, slipped from near the bottom of the stairs, and fell on the right side of the body. She was taken forthwith to the Hôtel Dieu, on the 4th April, 1823, complaining of pain in the arm and shoulder, the latter of which was severely bruised, and the former presented two slight wounds, one at the elbow, the other at the thumb. The acromion was prominent—the deltoid flaccid—a hard and irregular tumour was felt in the axilla, and, on rotating the humerus on its axis, motion and crepitus were felt. The evidence was conclusive—the bone was broken; and it only remained to employ the usual treatment. The limb was semi-flexed and placed upon a pillow—emollient cataplasms were placed upon the shoulder—and the patient was bled to two pallettes. The swelling had diminished next day, and the Baron, having directed extension to be made from the elbow, surrounded the arm with compresses; over them placed three splints upon the limb—one in front, one behind, and one upon the outside; and retained them in situ by a roller. The

---

by the bridle, when the animal suddenly throws up its head, the arm is converted into a lever, and the humerus is dislocated into the axilla. This is a common kind of accident and cases not unfrequently occur, where luxations, or even fractures, are produced, without the slightest direct fall or blow.

arm was semi-flexed and placed upon a pillow. On the 15th of May, the fracture was united, and the splints were taken off. On the 2d of June the patient was dismissed, with the perfect use of the shoulder and elbow-joints.

Sir Astley Cooper, in his Lectures, his larger and magnificent work on dislocations we cannot consult at this moment, observes, that in "fracture of the humerus, below its tubercles," the body of the bone "sinks into the axilla," a mark which the worthy Baronet gives as diagnostic. In the following case, the projection was not in the axilla, but the outside of the arm.

Case 2.—*Fracture of the Neck of the Humerus, with Displacement outwards of the lower Fragment.* Jean-Baptiste Biel, a shoemaker, 63 years of age, slipped down stairs, and struck his right shoulder against the bannister. He experienced very little pain after the fall, and, although he lost all use of his arm, he thought he had met with no serious injury, and merely applied a little bran-water to the bruise. He came to the Hôtel Dieu four days after the accident had happened. The right arm was hanging powerless by the side, and shorter than its fellow—the shoulder presented the marks of contusion, and the deltoid seemed thickened and shortened. Beneath this muscle was noticed an irregular and "bony" projection, which gave an unnatural and puzzling appearance to the part, and which quite disappeared on extending the limb to its proper length. On rotating the arm, the crepitus produced removed all doubt of the existence of fracture of the upper extremity of the humerus, although the projection of the end of the lower portion of the bone was not, as it usually is, in the axilla.

A cushion was fixed on the side of the chest by a bandage, the arm applied upon it, and retained in that situation by a roller, which also passed over the elbow. By this means, the arm was restored to its natural length, and the deformity of the shoulder disappeared, though the end of the bone projected somewhat still. The patient expressing inconvenience, the apparatus, on the 15th day, was removed, and a shorter cushion, thick below, employed in its stead. By this alteration the elbow was more tilted out from the side, and the upper end of the bone, of course, carried inwards, which reduced the projection almost to nothing. On the 25th day, the patient insisted on having the bandages &c. removed, and contented himself with placing his arm in a sling. The effects of his perverseness were pretty soon apparent, for a few days afterwards, in consequence of motion, the projection at the shoulder was as bad as ever. The arm was a second time bound to the side: at the end of eight days, the fracture was united; the projecting end of bone was no longer to be *seen*, (it could be felt); and forty-five days had expired since the accident, when the patient was dismissed from the Hôtel Dieu.

The projection, at the shoulder, of the end of the inferior portion of the bone, seems to us to have been caused by the action of the deltoid, which, as we are told in the report, was thickened and shortened. Would not properly-padded splints have been superior, in such cases, to merely binding the arm to the side? Sir Astley Cooper recommends the application of a roller from the elbow to the shoulder, and over it a splint, with proper pads on the inner and outer side of the arm. We know that in the thigh, where muscular contraction operates so strongly, the unyielding extension of Dessault's splint answers much better than any other method; and, looking at the result of the few cases we ourselves have witnessed, we should rather prefer the employment of splints, in fractures of the neck and upper end of the humerus.

The third case in the memoir before us is more complex than either of the former. The particulars are as follow.

An old soldier, 62 years of age, actually "in practice" as a cobbler, fell, whilst walking down a slope, on his left arm, which was closely applied to the side. He was received, next morning, in the Hôtel Dieu, and presented the following symptoms; considerable swelling of the shoulder-joint—shortening of the deltoid muscle, attended with increase of bulk, and a little flattening—unusual projection of acromion—impossibility of bringing the arm to the side—most obscure crepitus and motion of fragments—presence of a roundish prominence in axillâ, resembling a good deal the head of the humerus—and lastly, another projection beneath the tendon of the pectoralis major. Diagnosis was difficult, but M. Dupuytren decided for fracture. The apparatus adopted at the Hôtel Dieu, in this kind of injury, was applied,* but, two days afterwards, the swelling was augmented—the deltoid muscle less thick, less shortened, and more flaccid—the hollow beneath the acromion greater—the mobility diminished—and a rounded tumour presented itself in the axilla. M. Dupuytren, doubting the accuracy of his previous opinion, made extension of the limb, placed a cushion in the arm-pit, and bound the arm to the side by a circular bandage, the elbow being closely approximated to the chest, and advanced somewhat forwards and inwards, so as, of course, to direct the other end of the humerus upwards and backwards. In the course of five days the swelling disappeared, and then the real state of things was made out. A fracture was present, the fractured end of the lower portion of the humerus being felt unequal and rough in the axilla; but the head of the bone was also displaced, and carried something forwards and inwards. The bandages were continued, and at intervals renewed, till the fortieth day, when their use was abandoned altogether, the bone being united, and the limb of its ordinary length.

In a case of oblique fracture of the neck of the humerus, with displacement *forwards* of the lower extremity of the bone, the ordinary apparatus (described in the note) failed, in toto, in reducing the displacement. The arm was then laid upon a pillow, and several contrivances hit on to obviate the difficulty, but none had any very marked effect. At the end of a month, the displacement was not much removed, and the motion of the broken ends as great as ever. Strengthening diet, and the "vin antiscorbutique," were employed, and the fracture had united in another month: but still the lower portion projected in front to the extent of half an inch, or thereabouts.

The perusal of these cases then proves, that the diagnostic symptoms often vary, and are frequently obscure. The appearance mentioned by Sir Astley Cooper, viz. the projection of the end of the bone in the axilla, was absent in two out of four, and in both those instances the displacement was obstinate and never effectually removed. To recapitulate; our truest guides in these fractures are—the manner in which the accident occurs—the appearance of the shoulder and irregular form of the projection wherever that may be—and the crepitus produced on rotating the arm. We shall now consider some cases of luxation, in two of which the head of the bone was lodged in the axilla, in one it was dislocated backwards and outwards, and in one underneath the pec-

* This consists in applying a conical cushion to the axilla, and securing it there by a bandage. The arm is then bound to the side of the chest by several turns of a roller, some of which pass over the elbow, and the fore-arm is supported in a sling.

toral muscle. In both of the instances of dislocation downwards into the axilla, the fall had taken place on the palm of the hand, and the symptoms were marked and unequivocal. The hollow beneath the acromion, the slightly augmented length of the limb, the impossibility of raising the hand to the head, or bringing the elbow to the side, and the hard round tumour nearly filling the axilla, were characters so plain that he who ran might almost read them. The reduction was effected by seating the patient on a chair, placing a pad in the axilla to defend it from the pressure of the towel employed in counter-extension, one end of which passed in front of the chest, and the other behind it, whilst both were fixed to a ring in the wall. The extension itself was made from the *wrist* by means of a folded towel, applied in the form of a "grande bande," answering, we suppose, to the nautical *clove-hitch*,* which we use in this country. The wrist was defended from the pressure of the towel by a compress smeared with *cerate;* the reduction in both was readily effected.

The dislocation backwards happened in consequence of a quantity of earth falling down upon a man who was cleaning a sewer. It was readily known by the following symptoms:—violent pain in the joint, with incapability of moving it—elbow directed a little in advance—projection of the coracoid and acromion processes, as well as of the external part of the clavicle—visible depression and evident hollow beneath these points—and, lastly, prominence made by the head of the humerus behind and below the acromial apophysis. The head of the bone appeared to be situated at the external border and highest part of the fossa infra-spinata, an accident which Sir Astley tells us occurred only twice at Guy's Hospital in the space of eight-and-thirty years. The reduction, in the present instance, was effected by placing the patient in bed, making the counter-extension by the hands only of an assistant, from the "crest" of the scapula and axilla, and extension, also by the hands of two assistants, from the fore-arm and wrist. M. Dupuytren himself pressed the head of the bone towards its socket, and reduction was in this manner easily accomplished. The next case is one of a different description from any of the former, on which we have dwelt but lightly and briefly.

CASE VII. *Dislocation of the Head of the Humerus under the Pectoral Muscle—Reduction.*—Francis Hamlin, ætatis 26, a seal-engraver, whilst running along the roof of a house five stories high, in order to assist in extinguishing a fire, lost his footing, and fell to the ground, encountering and breaking in his descent a wooden shed. He was picked up insensible and carried to the Hôtel Dieu, when the house-surgeon discovered a dislocation of the right os humeri, and several severe contusions. The arm was placed upon a pillow, the bruises dressed, the patient bled, and on the following day he was seen by the Baron. He was lying on his back, with his arm upon a pillow directed somewhat backwards, and describing nearly a right angle with the body. The whole limb was greatly supinated, there was no unusual projection in the axilla, but under the pectoral muscles, and separated only a few lines from the clavicle, the head of the bone was distinctly felt. The patient could not raise the arm to the head, nor carry it backwards, and motion was attended

---

* Those who are ignorant of what the clove-hitch is, we would recommend to apply to some honest TAR. To attempt to explain it, would render the obscurum obscurius indeed.

with violent pain. On making him sit up, and forcibly bringing the elbow to the side, the acromion projected, and the deltoid was flattened, rigid, and shortened. It was evident to all that the head of the bone was lodged beneath the pectoral muscles, and having been bled once more to induce a state of faintness, he was taken to the operating theatre, and, after some trouble, the bone was reduced. At the end of twenty days he began to move the limb, but, in consequence of other injuries occasioned by the fall, he was not dismissed until after the lapse of two months.

This was an instance of complete dislocation forwards and upwards; but Sir Astley Cooper has described a case in which the dislocation is partial, the head of the bone not lying between the coracoid process and the ribs, but resting against its *outer* side. Sir Astley observes, that the accident is one of frequent occurrence, but we never ourselves have seen but one case, and that had a fatal termination.

Thomas Wood, 46 years of age, was hanging by his hands from the edge of the aperture of a trap-door in the roof, when suddenly the left hand slipped, and the weight of the body bore upon the right with a sudden and violent jerk. He did not fall, but the motion of the right arm was instantly lost, and the friends brought him into St. George's Hospital. The elbow stuck out from the side, and was directed rather backwards—the trifling elongation of the limb—the impossibility of bringing the arm to the side, or directing it forwards—the hollow beneath the acromion—the little or no numbness—and last, not least, the projection of the head of the humerus on the anterior part of the shoulder, plainly pointed out the nature of the accident. The house-surgeon, Mr. Lee, having made some ineffectual attempts at reduction, bled the man to thirty or thirty-six ounces, and gave him an ounce of the liquor antimonii tartarizati, which produced the greatest possible degree of syncope and sickness. At this instant, Mr. Lee taking hold of the elbow with one hand, the shoulder with the other, placed his knee in the axilla, and depressed the elbow, when the head of the bone glided into its socket with the greatest imaginable ease. The arm was then put up with a pad in the axilla, a figure of 8 bandage, and the arm was secured to the side. Next day the shoulder was easy, but the lancet-wound in the opposite arm was very sore, and the arm itself was stiff and uneasy. A black-draught and some common applications were ordered, and the house-surgeon, who had the immediate charge of the patient, being absent on leave, the circumstance, for a day or two, was little thought of. On the 19th September, three days after the bleeding, the arm was examined, and the neighbourhood of the wound was found to be swollen and hard, whilst the wound itself had inflamed and festered.

That night he was delirious, and next morning had a shivering and vomited his breakfast. The case now attracted serious attention, and at 2 P. M. of the 20th, we found him in the following state. His manner was hurried and approached to delirium, the pulse was 130, the skin burning hot, the tongue dry and brown, the abdomen distended, the bowels open. The whole of the arm was hard and swollen, the puncture was gaping, and from it a corded thickening was traced, exceedingly tender on pressure, in the course of the cephalic vein to the shoulder. The prognosis, of course, was extremely unfavourable, as acute phlebitis was established; a disease which, at the best, is pregnant with danger, in a London hospital almost uniformly fatal. The arm was poulticed, and salines with the tincture of opium prescribed, followed in the afternoon by a mixture containing carbonate of ammonia. On the 21st, there was little alteration in the symptoms, but towards the afternoon he vomited a quantity of greenish fluid, and was ordered some laudanum in hot brandy and water. The patient at this time presenting all the character of great depression of the vital powers, was directed to take half an ounce of the liquor ammoniæ acetatis, and an ounce of camphor mixture every four hours, together with opium and the submuriate of mercury. On the morning of the 22d, he appeared much relieved, but a second fit of shivering came on, and at 2 P. M. this was his condition.

The swelling of the arm had greatly subsided, no red streak was seen in the course of the cephalic, the pain upon pressure had entirely disappeared, in short the inflammation of the veins of the arm was decidedly passing away. His manner, however, was hurried and anxious, similar to that which those patients present who suffer from the formation of internal abscesses—he complained of some pain in the left side of the chest—tongue furred in the centre, red at the edges and tip—pulse very quick and indistinct, the slightest pressure commanding it entirely. The rigors, the peculiar delirium, the slight pain in the side, the characters of the pulse and the state of the tongue, indicated to all that insidious inflammation of the chest, of which so many cases had recently been seen at St. George's Hospital. The treatment consisted in the exhibition of camphor and opium, leeches to the abdomen, and a blister to the side, but matters very quickly got worse, and the patient expired on the morning of the 24th. No permission could be got to examine the body, but externally, the arm had regained almost its natural condition, the tint of the skin was yellow and jaundiced, the belly was tympanitic—the left side of the chest as dull on percussion as possible.

Having thus given cases illustrative of the symptoms of fracture of the neck and upper end of the humerus, as well as of the dislocations of the head of the bone, M. Dupuytren proceeds to detail several instances where the one kind of accident was mistaken for the other. This, as the most important and instructive division of the memoir, deserves to be attentively considered. The first case related is one of fracture of the neck of the humerus mistaken for a dislocation.

Catherine Godefroy, 60 years of age, was knocked down by the wheel of a cabriolet, and fell on her left elbow, separated to a slight extent from the body, and directed backwards. She fainted, and was carried to the house of a medical man, where she stopped two days, nothing being done throughout that time. Tortured with pain, and in despair at not recovering the motion of the limb, she desired to be taken to the Hôtel Dieu.

The shoulder at this time was swollen, and looking at the way in which the accident occurred, it became a question—what was the actual nature of the accident? The elbow was separated from the side, and any attempt at their approximation was attended with excruciating pain; a hard, round tumour appeared to present itself in axillâ; and the cellular membrane over the deltoid was injected with extravasated blood, which yielded on pressure and simulated that hollow beneath the acromion caused by luxation of the head of the bone. Under these circumstances, many believed that the accident was dislocation into the axilla. The patient was bled and cold lotions applied, but the Baron himself did not see her until the next morning. He pronounced it to be fracture of the head of the bone, and proved to all around that he was right. He shewed that though bringing the elbow to the side was very painful, it was easily accomplished, and remarked that the spheroid contour of th shoulder was preserved, the hollow being seated lower down. He felt the hard tumour in the arm-pit, but observed that it was higher than the head of the luxated bone, and on rotating the limb M. Dupuytren felt, and his pupils felt after him, evident crepitus. The nature of the injury was clear, and its treatment was simple. A wedge-shaped bandage in the arm-pit, in order to keep out the lower end of the bone which the pectoralis major and latissimus dorsi had drawn in—the lower end of the humerus drawn inwards and bound to the side by a bandage—the fore-arm bent, and fixed in the front of the chest. The case went on well, and the patient was discharged on the 19th of June, enjoying the perfect motion of the limb.

A patient presented himself to M. Breschet at the Hôtel Dieu, with the shoulder a little flattened—deltoid muscle rather flaccid—arm separated from the side, but readily made to touch it—limb shorter than the other—the projection of a fracture felt at the upper and inner part of the arm. The poor fellow applied for something to restore the motion of the limb, and disclosed the following tale. A year ago he had fallen from the top of a ladder on his shoulder, and a medical man pronounced that he had met with dislocation. Extension, &c. was made, the patient felt a crack, the luxation was thought to be reduced, and the arm was bound to the body. The displacement, however, returned though the motions of the limb did not. No doubt the case had been one of fracture, mistaken for dislocation, but a year had elapsed, when he saw M. Breschet, the mischief was past redemption's skill, and art could not repair after that lapse of time, the error committed at first.

The next case is curious as shewing the mistake of three surgeons in succession, and bears the following pithy title.

### Fracture of the Upper Extremity of the Humerus, three times mistaken for Luxation—three times reduced, and all to no purpose.

Joseph Nicholas Martin, a postilion, aged thirty four, entered the Hôtel Dieu, on the 5th of April, 1827. He had been knocked down by a horse on the 18th of January, and fell full on the left shoulder. A medical practitioner who saw him, said that the humerus was dislocated downwards, employed extension, and reduced a projection which existed on the anterior part of the shoulder. The arm, after this, was retained to the side of the chest for a month, when the patient being more tired of his doctor than the better for him, went to a second, and then to a third, each of whom enjoyed his pull at the "luxation." Joseph Nicholas Martin then entered the Hotel Dieu, in much the same state he was in at first, and shewing the following appearances.

The shoulder preserved its form and roundness, the acromion was not prominent, nor the deltoid flat. In front was a hard, and irregularly rounded tumour which followed the motion of the arm, and was evidently caused by the upper extremity of the lower portion of the fractured bone. Behind was a hard and resisting part, apparently the head of the bone, resting in the glenoid cavity, and covered by the posterior fasciculi of the deltoid, its anterior ones being stretched over the above-described prominence in front. The arm was shortened more than an inch, its motions difficult, rotation impossible. The hand could not be carried to the summit of the head, neither could the elbow be brought to the side.

That the case was one of fracture was plain, and that a serious mistake had been committed was palpable also. The patient, it would seem, had had his fill of the profession, for, in two or three days he left the Hôtel Dieu, refusing to submit to the trial of any apparatus.

### Dislocation of the Right and Left Humerus, and Fracture of the "Neck of the Femur," undiscovered.

Marie Jeanne Valletier, ætatis 48, whilst getting out of bed in the night of the 26th of January, for some necessary purposes, was seized with vertigo, and fell upon the left shoulder. She immediately felt violent pain in the part, and loss of the motions of the joint succeeded, but a surgeon to whom she applied next morning, having examined the shoulder, the arm, and the fore-arm, assured her that nothing was amiss, and applied a warm lotion to the seat of pain. On the 28th, however, the pain and incapability of motion continuing, she presented herself at the Hôtel Dieu, and then it was discovered, that the head of the humerus was lodged in the axilla. The deltoid was flattened, the acromion projected, and a hollow existed beneath it; a round hard tumour was felt in the axilla; the elbow was far from the side; and the patient could not raise the arm to the forehead.

Extension was made, and reduction effected, when M. Dupuytren, wishing to compare both arms, bared the other, and found it, much to his surprise, presenting the signs of dislocation forwards, under the pectoral muscle. The axilla was empty, the shoulder flat, the elbow stuck out, and directed backwards, and the head of the humerus lay on the front of the chest immediately below the collar-bone. Inquiry was made, and the following story elicited from the unfortunate patient. About twelve years had then elapsed since she had met with a fall down stairs, on the shoulder and hip of the right side. The medical men to whose care she fell, merely advised her to foment these parts, which she did, and kept her bed for full three months. At the end of this time she got up, and continued to get about with the aid of crutches, which, for the space of two years she was under the necessity of using. The motion of the arm she had never completely regained, and the lower extremity, on examination, was found two inches shorter than the other; the great trochanter was carried up near to the crest of the ilium; and the foot was turned out. These, of course, were now irremediable ills, and the patient who was maimed, through professional ignorance, in two of her limbs, and had narrowly escaped being visited with a similar curse in a third, was soon discharged cured from the Hôtel Dieu.

In a case of fracture of the upper extremity of the humerus, the éleve interne of the Hôtel Dieu, and through his means M. Dupuytren himself, in the first instance, were deceived. The individual was a woman, 70 years of age, who, whilst walking on some slippery ground, fell on the left shoulder, in which she immediately experienced a violent pain, but was able to regain her home, and thought she had merely experienced a bruise. At the end of three days, finding no relief, she applied at the Hôtel Dieu. The arm below was at this time much swollen, the deltoid muscle flat, the acromion somewhat prominent, the axilla filled up by a tumour which seemed to the éleve, the head of the humerus. Next morning M. Dupuytren, as we mentioned before, was at first deceived, but, on making a close examination, the greater mobility of the limb than luxation allows of, and other signs, demonstrated a fracture, or rather, a "crushing" of the head of the bone, with displacement of the upper extremity of the shaft in the axilla. A thick cushion was placed in the axilla, and the elbow bound close to the side, when all the deformity vanished, and the patient, on the 38th day was dismissed with the perfect motions of the limb.

The memoir concludes with two cases of fracture, mistaken for contusion, the first at a public hospital.

Julien Marcelin, 36 years of age, a mason, was thrown out of a cart, and fell on the left shoulder. Violent pain and incapability of motion succeeded, but on making application at the Hôpital Saint-Louis, he was told it was only a bruise he had met with, and ordered some leeches and a poultice. Tired at finding no relief, he applied at the Hôtel Dieu, eight days after the occurrence of the accident. M. Dupuytren grasping the elbow with one hand, and the shoulder with the other, rotated the arm, and instantly discovered by the motion and crepitus a fracture of the upper extremity of the humerus. The patient possessing a willing and docile disposition, the treatment employed was situation alone. The limb was placed in a semi-flexed position, a little apart from the body, on a pillow, and held there by two bands, the one attached to the arm, and maintaining the portions of the bone in apposition, the other fastened above the wrist to prevent the motion of the arm. This position was maintained for twenty-five days, at the end of which time, the fracture was united without deformity, and the arm was transferred to a sling.

Eugéne Maxime, ætatis 49, a toy-man, fell on the left shoulder, in which he immediately felt great pain, and lost the motions of the arm; considerable swelling ensued, and a surgeon being consulted, pronounced it a violent contusion, and ordered some common applications. No amelioration taking place, the man applied at the Hôtel Dieu eight days after the accident, with the marks of a bruise on the shoulder, but neither deformity nor shortening of the limb. On employing rotation from the elbow, a tell-tale crepitus discovered the mistake that had been made in diagnosis, a mistake which the swelling of the parts at the time, and the want of displacement combine, we should think, to render venial. The treatment employed was similar to that had recourse to in the last-mentioned case; the fracture was united perfectly in place, and the patient was dismissed on the 36th day.

This brings to a close the memoir, of which we have given an analysis. M. Dupuytren's remarks, we think, are essentially confirmed by his cases, and his cases are instructive examples of surgical ignorance detected and corrected by surgical skill. Perhaps it may appear that the errors related are unlikely to occur in any who possess but a moderate knowledge of their craft, and are, therefore, to be viewed as a source of amusement, rather than as one of practical importance. Would to Heaven that this were really the case, that none but the ignorant or careless could err, that the able and attentive and scientific surgeon could never be mistaken, could never be open to the whispering insinuations of the jealous rival, or the devilish and undisguised attack of the reckless libeller! Errors in diagnosis are the lot of all, and the difference between the skilful and unskilful surgeon is this—not that the former is free from blunders, but that *he* makes one to the other's twenty. We remember having witnessed, a little time ago, the case of a patient, who, some six weeks or two months before, had received an injury about the shoulder. Nothing, we believe, had been done by his medical attendant, and the patient, at the end of the time above-mentioned, consulted a surgeon, who deservedly ranks amongst the highest in this town at the present day. The acromion projected, the deltoid was flattened, the arm was lengthened, its motions were lost, a tumour of some kind was felt in the axilla, every thing, in short, seemed to indicate luxation of the head of the humerus into the axilla, and every one present considered that such was really the case. Under these circumstances, the pullies were employed to reduce the dislocation, and after some time, the reduction did actually seem to be accomplished, when the straps and the other paraphernalia were removed. A very few minutes, however, had elapsed, when the symptoms of luxation had all returned, the shoulder was as flattened as before, and the head of the bone appeared to have relapsed to its former situation. A second reduction was attended with a similar result; and it then became a question, which, up to this moment, no one can answer, whether the case was one of dislocation or fracture of the upper extremity of the bone. It is but a few days ago that we saw a young gentleman, the son of a distinguished nobleman, who had met with a fracture of the neck of the scapula, by a fall from his horse, which fracture had not been suspected by the surgeon, to his no small disgrace in the eye of his patient.

We mention these cases to shew that the subject is one which deserves, and we hope will receive, due attention from our surgical brethren. We know of no paper in which the diagnostic marks of luxation and fracture at the upper extremity of the humerus, are so fully laid down as in that of M. Dupuytren's; and so far as it goes, we believe it to be a valuable contribution to the knowledge we possess of these particular accidents. At the present day, when brother practitioners are lynx-eyed to each other's faults, when one little slip of the head or the hand is dragged before the public, the non-professional public, with malignant glee, and wrought by stipendiary scoundrels into some tale of "butchery and blood;" at such a time, we say, it behoves us all to be well upon our guard, and glean from all quarters that useful information which must prove, after all, our most effectual defence. If the routine practitioner, who blindly trudges on, like an ass in a mill, the same unvarying and beaten round, considers such minutiæ as troublesome and useless, the retribution will fall upon his own head at last. If this, indeed, were all, it would not matter much; but the mischief of it is, that such an individual is a curse to the community, as well as a foe to himself. The number, however, of those who set their faces against improvement, too lazy to read, too obstinate to learn, is rapidly decreasing, and better-informed men will speedily rise, like Banquo's ghost before the usurper, to "push them from their stools."

